# A Wireless Sensor Network-Based Ubiquitous Paprika Growth Management System

**DOI:** 10.3390/s101211566

**Published:** 2010-12-16

**Authors:** Jeonghwan Hwang, Changsun Shin, Hyun Yoe

**Affiliations:** School of Information and Communication Engineering, Sunchon National University, Maegok-dong, Suncheon-si, Jeollanam-do, Korea; E-Mails: jhwang@sunchon.ac.kr (J.H.); csshin@sunchon.ac.kr (C.S.)

**Keywords:** wireless sensor networks, ubiquitous, agriculture, greenhouse, paprika

## Abstract

Wireless Sensor Network (WSN) technology can facilitate advances in productivity, safety and human quality of life through its applications in various industries. In particular, the application of WSN technology to the agricultural area, which is labor-intensive compared to other industries, and in addition is typically lacking in IT technology applications, adds value and can increase the agricultural productivity. This study attempts to establish a ubiquitous agricultural environment and improve the productivity of farms that grow paprika by suggesting a ‘Ubiquitous Paprika Greenhouse Management System’ using WSN technology. The proposed system can collect and monitor information related to the growth environment of crops outside and inside paprika greenhouses by installing WSN sensors and monitoring images captured by CCTV cameras. In addition, the system provides a paprika greenhouse environment control facility for manual and automatic control from a distance, improves the convenience and productivity of users, and facilitates an optimized environment to grow paprika based on the growth environment data acquired by operating the system.

## 1. Introduction

The term wireless sensor networks (WSNs) refers to a widely used technology used for surveillance and control whereby sensor nodes equipped with computing and wireless communication capacity are placed in various application environments, forming a network that collects and distributes by radio information acquired from sensor nodes [1,2]. WSN technology is one of the technologies which are important to realize the so-called ubiquitous society, which can be applied to various industries such as distribution, logistics, construction, transportation, national defense, and medicine, to achieve advances of productivity and safety, and improve the quality of human life [3].

Agriculture in particular, is labor-intensive compared to other industries, and when WSN technology is applied to the agricultural area, which is generally lacking IT technology applications, added value and the productivity of agriculture can be increased [4,5]. Recently, various studies exploring the use of WSN technology in protected agriculture environments such as greenhouses, stables and precision farming have been conducted [6,7], and advanced nations including E.U. and the U.S. have already established monitoring systems in areas such as cultivation environment monitoring and production management and distribution in order to improve the production of agricultural and stock breeding products and facilitate distribution route transparency [7,8]. Additionally, cutting edge scientific farming experiments have been realized in several areas of the horticulture industry in advanced nations such as The Netherlands and Japan [9].

Compared to more advanced nations in this field, Korea lacks research integrating agriculture and IT technologies as well as tools to collect growth environment information and analyze monitoring data, and its agricultural output costs are typically high but the output is low, because the level of technology optimized for environmental control is low compared to some of the more advanced nations [10]. In the concrete case of paprika, Korean paprika output per unit area is only 30% that of The Netherlands [9], which is an advanced nation in the horticulture field, due in part to a lack of technologies for monitoring different types of cultivation and growing environment management. Consequently, to improve the quality and increase the yield of paprika products, a real-time growth environment monitoring and control system on the whole greenhouse system is desirable.

In response to this problem this study designs and realizes a ‘Ubiquitous Paprika Growth Management System’ which makes it possible to monitor the environment used to grow paprika and manage and control this growing environment in real time using WSN technology, thus providing high quality paprika cultivation greenhouses with a precise growing environment.

The proposed system improves productivity by maintaining an optimized environment for growth and development through information about the environment in the paprika greenhouses and the growth and development of the crop, and it not only reduces production costs by optimizing management of the production components but also provides convenience to users through automatic wired-wireless remote control of the paprika growth environment.

The structure of this article is as follows; Section 2 explains related research, Section 3 describes the structure of the proposed ubiquitous paprika greenhouse management system structure and provided service processes, and Section 4 presents the results of our implementation of the proposed system. Finally, the conclusions that sum up the paper are presented in Section 5.

## 2. Related Research

Serodio *et al*. developed and tested a distributed data acquisition and control system for managing a set of greenhouses. Several communication techniques were used for data communications. At a lower supervision level, a WLAN network with a radio frequency of 433.92 MHz was used to link a sensor network to a local controller inside each greenhouse. A controller area network (CAN) was provided to link an actuator network to the local controller. Through another RF link (458 MHz), several local controllers were connected to a central PC. High level data communication through Ethernet was provided to connect the central PC to a remote network [11,12].

Morais *et al*. have implemented a wireless data acquisition network to collect outdoor and indoor climate data for greenhouses in Portugal. Several solar-powered data acquisition stations (SPWAS) were installed to measure and monitor the data. RF links were established among multiple (up to 32) SPWASs and a base station, which was used to control the SPWASs and to store the data [13].

Liu and Ying have reported a greenhouse monitoring and control system using the Bluetooth technology. The system collected environmental data from a sensor network in a greenhouse and transmitted the data to a central control system [14]. Mizunuma *et al*. deployed a WLAN in a farm field and greenhouse to monitor plant growth and implemented remote control for the production system. They believed that this type of remote control strategy could greatly improve productivity and reduce labor requirements [15].

The Rinnovando group is doing research work in a tomato greenhouse located in the South of Italy. They are using a wireless sensor network with Sensicast devices for the air temperature, relativity humidity and soil temperature measurements. They have also developed a Web-based plant monitoring application. A greenhouse grower can read the measurements over the Internet, and an alarm will be sent to his mobile phone by SMS or GPRS if some measurement variable changes rapidly. The Rinnovando group has installed a test bed in a 20 × 50 m tomato greenhouse, where six nodes are deployed into two rows 12.5 m apart from each other. One mesh node works as a repeater and improves the throughput of the communication. A bridge node gathers data from other sensor nodes, which transmit the measurements of temperature and relative humidity in one minute intervals [16].

Yoo *et al*. have described their results on a real deployment of a IEEE 802.15.4 compliant WSN to monitor and control the environment in greenhouses where melon and cabbage were grown [17,18]. Lea-Cox *et al*. have developed a greenhouse WSN that integrates a variety of sensors which can measure substrate water, temperature, electrical conductivity, daily photosynthetic radiation and leaf wetness in real-time. Benefits came from an improved plant growth, more efficient water and fertilizer applications, together with a reduction in disease problems related to over-watering [18,19].

Liu *et al*. have developed and tested a WSN prototype for environmental monitoring inside a greenhouse using a star topology network of Crossbow MICAz motes. The motes measure temperature, humidity and soil moisture, and send their measurements to the sink node in five minutes intervals. The sink node is a combination of a MICAz mote and a MIB510 board with a data terminal. A terminal with an ARM processor module shows the lastest measurements on a LCD-screen inside the greenhouse and delivers the data to the main PC by using a GSM module. The central PC located remotely from the network takes care of data logging and processing. Mote programming and data receiving is possible through the RS-232 serial interface provided by the MIB510 board. The Received Signal Strength Indicator (RSSI) values over the distance between nodes with different antenna heights and polarization angles were compared to each other. Based on the results it was possible to conclude that the longest communication range was achieved when nodes had the same orientation and maximal antenna height. The temperature difference in experimental measurements between two nodes, where one node was placed in the center of the greenhouse and another near the greenhouse wall indicated the existence of the microclimate layers [20].

Zhou *et al*. designed a monitoring system based on ZigBee, using an star network topology inside the greenhouse and a mesh topology for the connection between the greenhouses and the management system [18,21]. Yang *et al*. reported a multi-functional remote sensing system that integrates RFID technology with spectral imaging and environmental sensing in a greenhouse. The multi-spectral imaging system was used for remote sensing of the canopy of cabbage seedlings. Greenhouse temperature, relative humidity, and lighting conditions were measured above the crop [22]. Finally, Wang *et al*. have developed a specialized wireless sensor node for monitoring temperature, relative humidity and light inside greenhouses [23].

## 3. Design of the Ubiquitous Paprika Growth Management System

### 3.1. Paprika Growth Management System Structure

**Figure 1.**
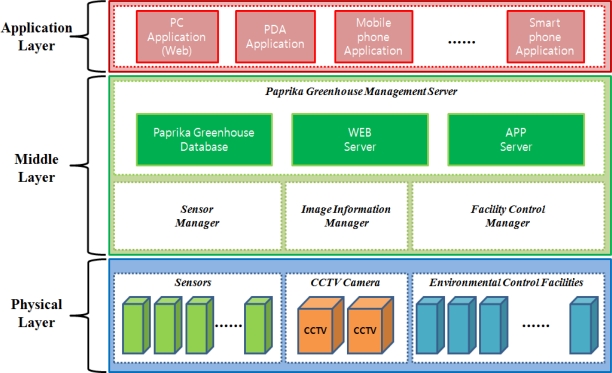
Paprika Growth Management System Structure.

The proposed ubiquitous paprika greenhouse management system is composed of three layers as shown in Figure 1. The layers include physical, middle and application layers.

The physical layer is comprised of sensors, CCTV cameras, and environmental control facilities. The application layer includes facilities for monitoring paprika growth and the greenhouse environment information and the interface supporting services for growth environment control, and the middle layer supports communication between the physical and application layers and maintains the optimized conditions for growth and development of crops through a database for the greenhouse information and by providing monitoring and control services.

#### 3.1.1. Physical Layer

The physical layer is composed of sensors collecting information about the external and internal environment of the paprika greenhouse and the growth of the paprika, the CCTV cameras collecting image information on the greenhouses, and environment control facilities to create an optimized environment for paprika growth.

Sensors are broadly divided into environment sensors collecting information from the internal and external environment and growth sensors collecting information on the growth of the plants. Environment sensors measure a variety of parameters which affect the growth of paprika such as intensity of illumination, temperature, humidity, wind direction, wind speed EC, pH, and CO_2_ levels, *etc*. Growth sensors measure changes in the growth and development of paprika plants such as the temperature of leaves and parts of stems, plant body weight and height, fruit temperature and volume.

The CCTV system is installed inside and outside of greenhouses. The internal CCTV is for collection of image information on the paprika and the external one for prevention of events such as burglary and fires.

Environment control facilities include ventilation and heating systems that can control the various greenhouse environment factors that affect the growth of crops such as illumination, temperature, EC, pH, and CO_2_, systems to retain heat for reduction of energy consumption, systems of controlling curtains to shade the light according to its intensity, circulating fan systems to control the circulation of air inside of facilities, systems to the control temperature of hot water and working fluids, and systems to control an artificial source of light according to the external light intensity, and each environment control facility is controlled by a PLC (Power Line Communication).

#### 3.1.2. Middle Layer

The middle layer is comprised of the sensor manager for management of environmental information collected by sensors, the image information manager for management of image information collected from the CCTV system, the facility control manager for management of the environment control facility, a database for storing paprika greenhouse information, and the paprika greenhouse management server for control of the paprika greenhouse monitoring and environment control facility.

The sensor manager processes formats in the form that information about the environment outside and inside greenhouses and about paprika growth, which are collected from the physical layer sensors, can be saved in the paprika greenhouse database, converts it into units which are suitable for the measurement elements and saves the processed data in the database using updating inquiries.

The facility control manager operates or the manages environmental control facility through PLC by receiving control signals from the paprika greenhouse management server, and conditions of such a environmental control facility, operation time and the number of controls are saved in the paprika greenhouse database.

The image information manager provides streaming data to the web by transferring images from the CCTV system to the paprika greenhouse management server, and saves it to the paprika greenhouse database after classifying it by greenhouse ID and camera number.

The paprika greenhouse database saves data in each table, including greenhouse environment data such as intensity of illumination, temperature, humidity, and CO_2_, which are collected from the sensors installed in the internal and external environments of the paprika greenhouse, the temperature of leaves and parts of stems, growth and development data such as weight of plant bodies, fruit temperature and volume, image data collected from the CCTV system, conditions, operation time, and number of controls of the environmental control facility, automatic control and standard values for condition notification.

The paprika greenhouse management server is located between the users and the paprika greenhouse database, and automatically controls all the environment control facilities and provides an alarm service through the web or SMS by informing users of greenhouse environment data and growth data saved in paprika greenhouse database over a certain period and comparing standard environment values saved in environment control facility control table and the condition alarm table.

#### 3.1.3. Application Layer

The application layer is composed of application services supporting various platforms such as web, PDA, smart phone, *etc*. which can provide users with paprika growth information monitoring services, greenhouse environment monitoring services, greenhouse image monitoring services, paprika growth and development environment control services.

### 3.2. Paprika Growth Management System Services

#### 3.2.1. Paprika Growth Information and Greenhouse Environment Monitoring Services

Paprika growth information and greenhouse environment monitoring services save paprika growth and development information such as information of internal and external environment of paprika. This service is broadly divided into crop growth information services, greenhouse internal and external weather information services, and rooting zone environment information services. Crop growth and development information services measure and analyze temperature of plants inside of paprika greenhouses, the temperature of the upper and lower parts of stems, temperature and volume of fruits, body weight of plants, plant heights, rates of increase of weight and height of the plants, the amount of water and light that crops absorb, yields, *etc*.

Greenhouse internal and external weather information services measure and analyze external weather conditions such as temperature, humidity, wind direction and speed and internal weather such as temperature, relative humidity, the intensity of light for the upper and lower parts of crops, and the amount of light penetration. Rooting zone environment information services analyze and provide producers with information on measurements and change of conditions such as the amount of supplied liquid affecting the rooting zone of plants inside of greenhouses, EC and pH of supplied liquid, the amount, EC, and pH of waste liquor, rate of absorption and temperature within culture medium, and temperature of supplied water and waste liquor.

**Figure 2.**
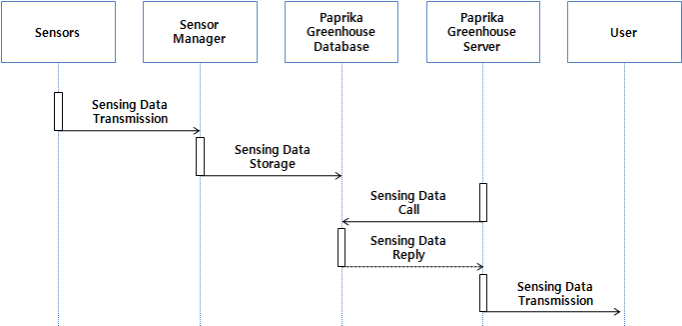
Paprika Environment & Growth Information Monitoring Operations.

At this point, the sensor manger analyzes the delivered data, extracts and transforms each value of sensing, and saves in each table of the paprika greenhouse database. The paprika greenhouse management server then delivers this information about the greenhouse internal and external environment and growth and development of paprika saved in paprika greenhouse database, and through this the users can monitor information on the growth and development of the paprika plants and the greenhouse environment. Figure 2 shows the process of operation of the monitoring services for the paprika growth and development and the greenhouse environment.

#### 3.2.2. Paprika Image Monitoring Services

Image monitoring services for paprika greenhouses are to provide producers and consumers with images of greenhouses and crops through CCTV installed inside and outside of greenhouses.

**Figure 3.**
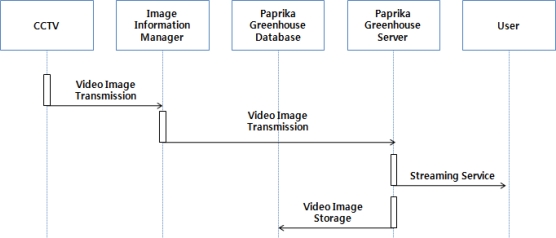
Paprika Image Monitoring Operations.

Images collected from the CCTV system are sent to an image information manager that classifies this stream of data by greenhouse ID and camera number and saves it in the paprika greenhouse database, and transmits it to paprika greenhouse management server and manages matching between ID and image information, which enable users to check image information of greenhouse through web server. Figure 3 shows operation of the paprika image monitoring services.

#### 3.2.3. Paprika Growth and Development Environment Control Services

**Figure 4.**
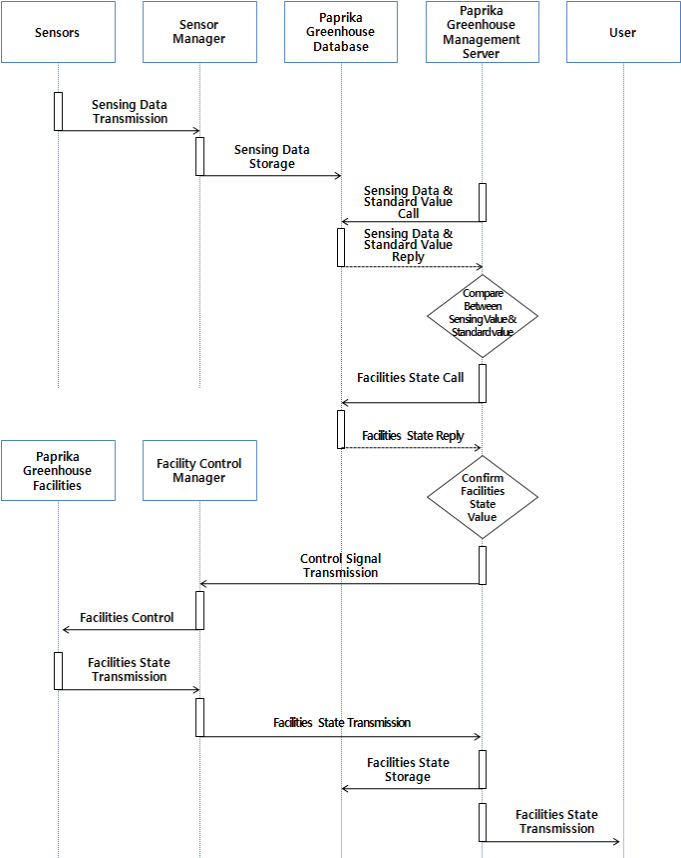
Paprika Facilities Automatic Control Services Operations.

Paprika growth and development environment control services can is controlled automatically by the paprika management server or manually by producers for creation of an optimized environment for growth and development of paprika based on information collected from sensors installed inside and outside of greenhouse and the CCTV system. Figure 4 illustrates the operation process of the automatic control services of the paprika environment control facilities.

Automatic control services save information on the environment and growth and development of paprika collected from greenhouses, and the paprika greenhouse management server calls this information and compares the collected information with the standards saved in the paprika greenhouse database. After checking whether the environment control facilities saved in paprika greenhouse database match or not the values set in the standards, control signals are sent to the facility control manager and the facility control manager transmits the control signals with PLC to control each environment control facility.

**Figure 5.**
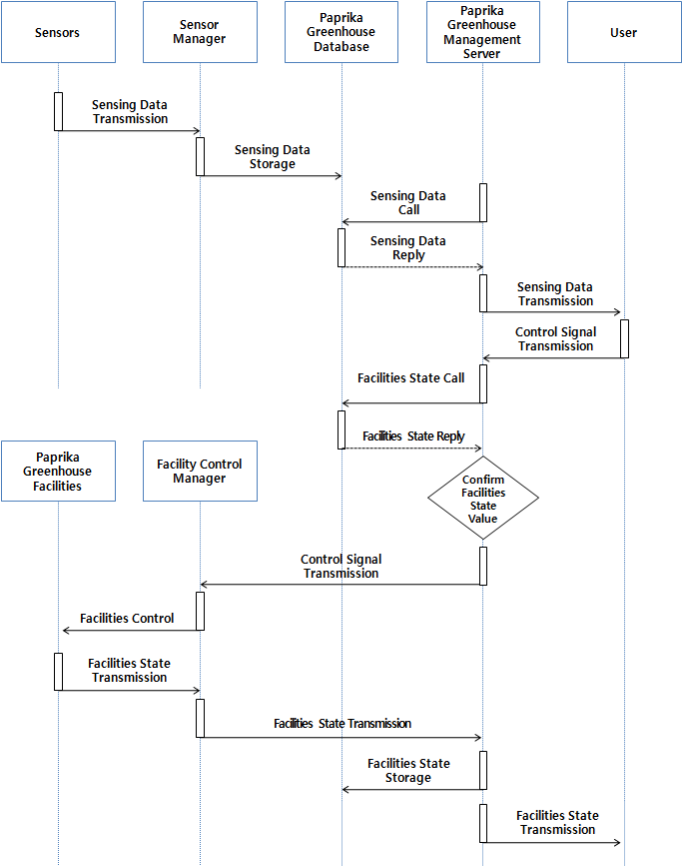
Paprika Facilities Manual Control Services Operations.

When the environment control facility operates, information on the conditions, operation time, and number of control of environment control facility is saved in paprika greenhouse database through the facility control manager and users are informed through the GUI. Figure 5 illustrates the manual control services of the paprika environment control facilities.

Manual control services save information about the environment and growth and development of paprika collected from greenhouses in the paprika greenhouse database and the paprika greenhouse management server transmits this information to users in real time. Users can check the information about the environment of the greenhouses and the growth and development of the paprika through the GUI, and when there is a need to use the environment control facility, a facility control order is given through the GUI, which receives facility control orders from users and transmits facility control signals to the paprika greenhouse management server, and paprika greenhouse management server that receives the control signals understands whether environment control facility is operating correctly or not from the paprika greenhouse database, and transmits appropriate control signals to the facility control manager and controls the environment control facilities through the PLC.

#### 3.2.4. Paprika Greenhouse Status Alarm Services

**Figure 6.**
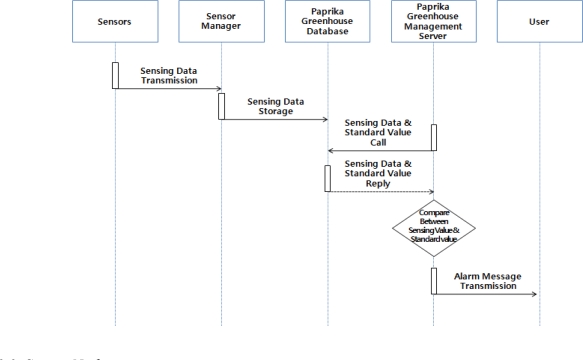
Operation process of the Paprika Greenhouse Status Alarm Services [18].

Paprika greenhouse situation alarm services serve are to prevent dangerous situations by informing users of changes in weather and greenhouses conditions and letting them take measures in advance. Environment sensors transmit the measured data to the sensor manager. The sensor manager extracts the measurement values from the transmitted data and saves it in paprika greenhouse database. The saved data is periodically monitored by the paprika greenhouse management server, and when the values are over or under the standards, or environment changes occur, the users are informed through the web or by SMS (Figure 6) [18].

### 3.3. Sensor Nodes

**Figure 7.**
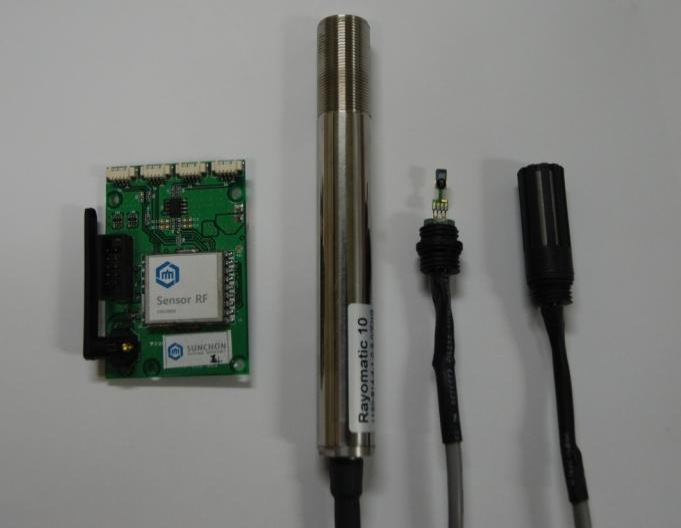
Sensor Node.

The sensor nodes used in this system receive with the greenhouse and leave temperature and humidity sensor data from the corresponding sensors via a MSP430 MCU and transmit it to relay nodes and the gateway using a CC2420 RF Chip [24]. In addition, in order to minimize the effects of the heat generated in nodes, nodes and sensors are separated. Since the MSP430 as a 16 bit RISC has 48 Kbytes of program memory and 10 Kbyte RAM inside, multiple sensor data can be dealt with at a high speed, and the CC2420 supports 2,400–2,483.5 MHz band width with an RF Chip supporting ZigBee, and operates in DDDS mode, making possible real-time wireless communication with low electricity consumption by supporting the modulation method of O-QPSK and a 250 Lbps Data Rate. Figure 7 is the sensor node for measuring environment of paprika greenhouse, and in order to apply it to a wide range of sensor networks, it uses small sized sensor nodes with low energy and cost.

#### 3.3.1. Power Supply

The power for sensor nodes is provided by using a TEKCELL 5.6 V battery, and a TK71750 LDO is used for supplying stable power to the nodes [25]. The LDO is composed of a pass transistor and error amplifier; the pass transistor plays a role as a voltage-controlled current source like a PNP TR or PMOS, and the error amplifier maintains a constant voltage regardless of change of pressure voltage or output load, by receiving feedback from the output [24].

#### 3.3.2. Temperature and Humidity Sensors

As temperature and humidity sensor, the all-in-one style SHT71 is used. The operation power is 2.4 V–5.5 V, which is relatively low, and power consumption is as low as mean 28 μA. The inside of the sensor has offset memory, 14 bit A/D converter and digital 2-wire interface, and the temperature can be measured up to −40 to 120, with an accuracy of 0.5%. In addition, humidity is measured between 0 and 100% with an accuracy of 3.5% [26]. A 3.3 V operating voltage is connected to sensor nodes and by connecting a digital 2-wire with the MSP430 circuitry, the greenhouse temperature and humidity information is dealt with [24].

#### 3.3.3. Leaf Temperature Sensors

The sensor used for the temperature of leaves is a IRtec Rayomatic 10. This is a suitable sensor to measure the surface temperatures like that of the surface of leaves since it is a non-contact smart infrared light sensor that can measure a wide range of temperatures between 0 and 500 °C. The Rayomatic 10 sensor for leaf temperatures uses signals by amplifying them with a LM358 OP-AMP in order to gain the correct value of sensor, since the current change of current according to the temperature of leaves is as small as 4–20 mA [24].

#### 3.3.4. Leaf Humidity Sensors

As a leaf humidity sensor a Model 237 Leaf Wetness Sensor was used, which changes the values of internal resistance, which is Rs, in between 20 and 1,000 KΩ according to the wet or dry conditions of the surface of the sensor and can measure the humidity of the surface of leaves between 0 and 100%, according to the values of the internal resistance changes. Since the magnitude of signal change of the sensor according to the changes of internal resistance is minute, it is used together with a LM358 OP-AMP like the temperature of leaves sensor in order to determine an exact value [24].

#### 3.3.5. Controller

The MSP430 is a 16 bit RISC MCU, whose operating voltage is between 1.8 V and 3.3 V. There are two external clocks and one internal clock can be generated by itself. A XIN-XOUT pin is a connection place with a low frequency clock, which is used to operate into low energy mood, and XT2IN-XT2OUT pin is a place of connecting with high frequency clock, which is for operation of high performance mood. In addition, the strength of MSP430 is that it has 12 bit built-in ADC with eight channels so it can use digital data by directly changing analogue signals entering from sensor to digital data without composing extra ADC. There are two types of timer; Timer A and Timer B. Timer A has three CC registers, and Timer B has seven registers. Practical timer has two registers, but because it has register, it can have effects of several timers. In addition, there are two UARTs with six 8 bit ports. Besides, it is MCU with built-in hardware multi-player and DMA controllers.

MSP430 receives 3.3 V voltage from the power supply, calculates sensor data by receiving signals from sensors for temperature, humidity, temperature and humidity of leaves, and sends it to CC2420 RF Chip. In order to use with low power mode, it connects the XIN-XOUT 32.768 Khz Crystal generator to the XIN-XOUT pin [27].

#### 3.3.6. Wireless Communication

CC2420 is 2.4 GHz of ZigBee chip, which is suitable for low power and low cost sensor networks. It uses the O-QPSK modulation method by applying DSSS and Half Sine Pulse. The maximum transmission rate is 250 Kbps. In addition, it has four serial ports including SI, SO, SCLK, and CSn, as well as thirty-three 16 bit composition and condition registers, fifteen order saving registers, two 8-bit transmission and reception FIFO registers. The CC2420 connects through the MCU and 4-wire SPI interface and approaches 128 bytes of FIFO related to transmission and reception [28].

## 4. Implementation of the Ubiquitous Paprika Growth Management System

**Figure 8.**
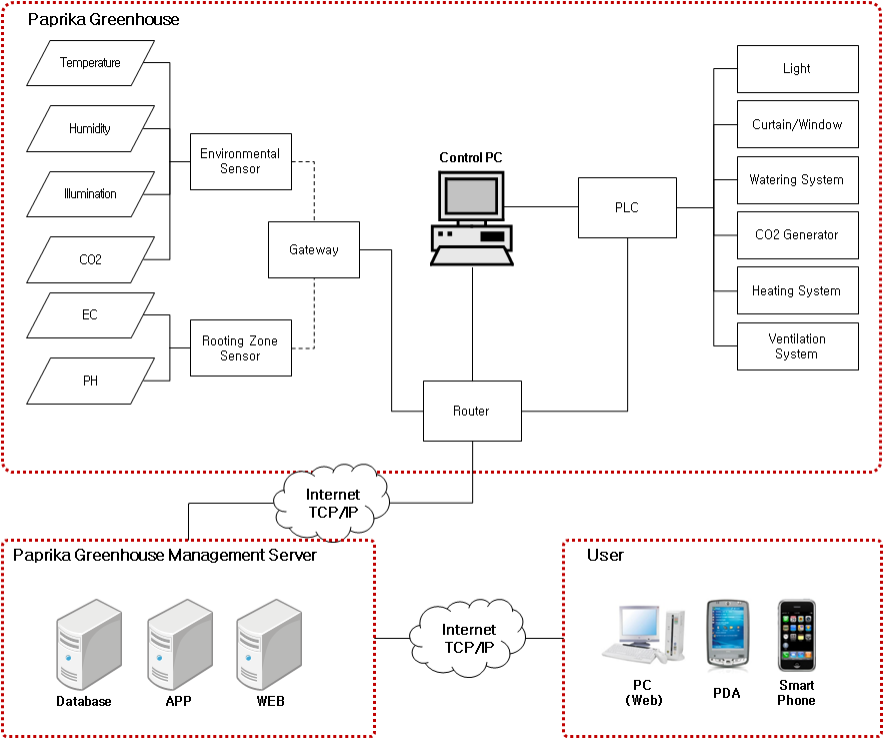
Block Diagram of Proposed Ubiquitous Paprika Growth Management System.

In order to prove the utility of the proposed ubiquitous paprika growth management system for growth and development of paprika, firstly, this study established a system representative of an actual paprika greenhouse after testing the systems by installing sensors, including sensors for soil, environment, temperature and leaf humidity in paprika greenhouse models. Figure 8 is the block diagram of such a ubiquitous paprika greenhouse system, the installed environmental sensors and rooting zone sensors in paprika greenhouse collect environmental information and paprika growth information. The CCTV system is installed inside and outside of paprika greenhouses and collects paprika and paprika greenhouse images. The paprika greenhouse has environment control facilities that can control the greenhouse environment features which affect the growth of crops such as light, curtains, windows, watering systems, CO_2_ generator, heating systems and ventilation systems, and each environment control facility is controlled by PLC.

Users can monitor the paprika greenhouse and control facility information via the “Control PC” installed in paprika greenhouse. The paprika greenhouse management server is composed of the paprika database, WEB server and APP server, so users can thus monitor and control the paprika greenhouse using various types of devices such as PCs, PDAs, smart phones, *etc.*

### 4.1. Test-Bed: Greenhouse Model

**Figure 9.**
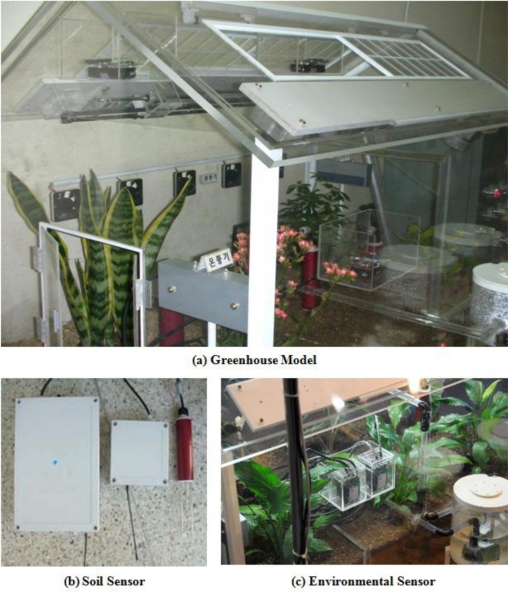
Test-bed: greenhouse model and sensors.

In order to prove the feasibility of the system, paprika greenhouse models were produced, as seen in Figure 9, and soil, environment, and temperature and humidity of leaves sensors were installed for monitoring of the environments of these model greenhouses and the growth and development of crops, along with a CCTV for monitoring of images of outside and inside greenhouses.

**Figure 10.**
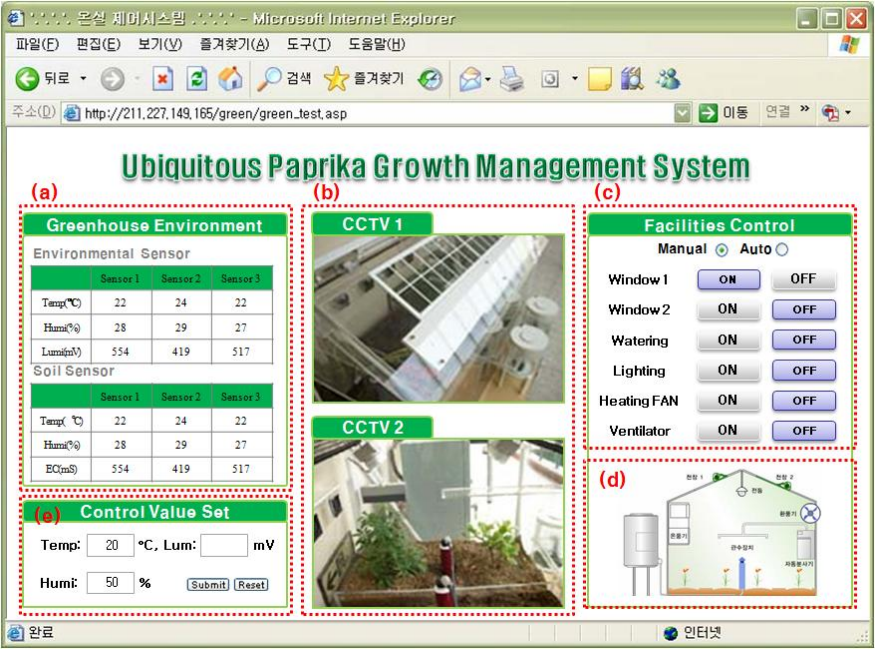
Test-bed GUI.

In addition, in order to maintain an optimized environment for growth and development of crops, electric lights, fan heaters, ventilators, devices for overhead flooding, and ceilings, environment control facilities are installed in greenhouses, and in order to test system control and monitoring, the Web GUI of the ubiquitous management system for growth and development of paprika was realized, as seen in Figure 10.

The values which were measured by the soil and weather sensors in greenhouses are viewed in window (a) and from (c), we can control the greenhouses facility devices and check conditions. The paprika greenhouse management server compares the sensing values with those stored in the paprika greenhouse database. When the value of the conditions of an equipment device is 1, it appears as “Onm”, and when it is 0, as “Off”. Window (d) displays equipment conditions in (c) as graphics. Window (b) show the images collected through the CCTV cameras, and (e) is where the standard values for automatic control of the greenhouse are entered.

### 4.2. Field Application: Paprika Greenhouse

**Figure 11.**
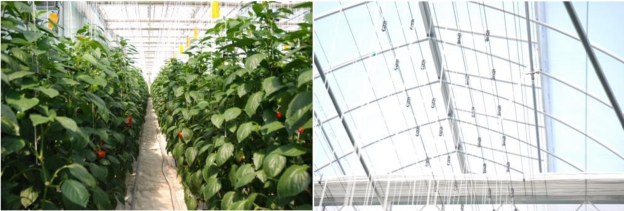
Installation site of the Ubiquitous Paprika Growth Management System: paprika greenhouse in Sepung-ri, Gwangyang-eup, Kwangyang-si, Jeollanam-do, Korea.

As a result of applying the proposed ubiquitous paprika growth management system to greenhouse models, the usefulness of system was confirmed, and systems were established to test its use in actual paprika greenhouses, as seen in Figure 11.

In order to gather information about the internal and external environment of the paprika greenhouse, sensors were installed inside and outside, and internal and external features such as temperature and humidity outside and inside the greenhouse, wind speed and direction and internal greenhouse parameters such as temperature, relative humidity, intensity of light in the upper and lower parts of crops, and the amount of penetration of light.

**Figure 12.**
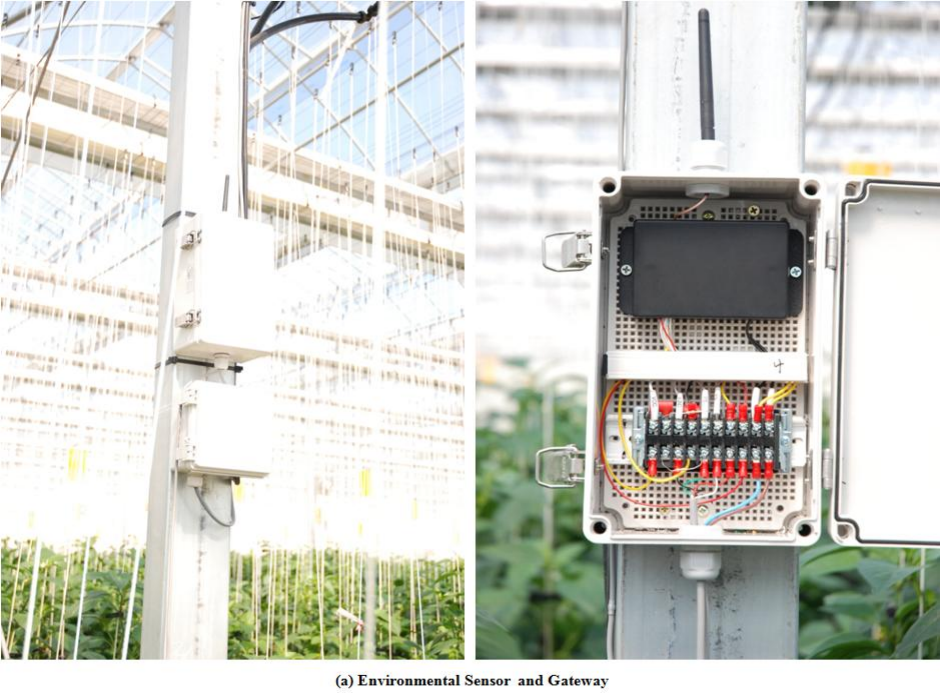

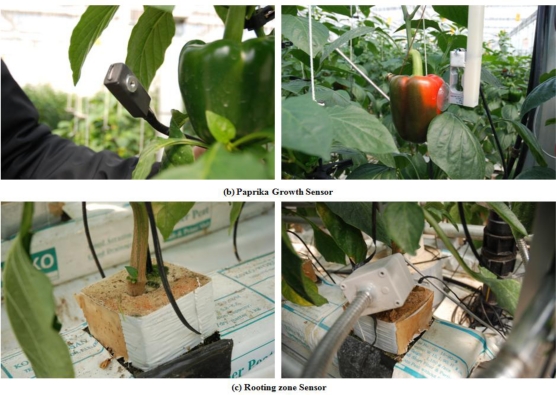
Installed Sensors in the paprika greenhouse.

In addition, considering that paprika cultivation mainly uses nutrient solution culture, management of the rooting zone, which greatly affects absorption in nutrient solution culture, is very important, and factors such as the amount of nutrients, EC and pH of supplied liquids, the amount, EC, and pH of waste liquors, rate of absorption and temperature within the culture medium, and temperature of water supply and waste liquor, were measured by installing sensors to collect information about the rooting zone environment. In order to understand the growth and development of the crop, sensors were installed and the temperature of plants inside the paprika greenhouse, the temperature of the upper and lower parts of stems, temperature and volume of fruits, weight of plant bodies, plant body heights, the amount of water and light that crops absorb, yields, and rates of increase of plants body weight were measured. Figure 12 shows installed sensors in the paprika greenhouse.

**Figure 13.**
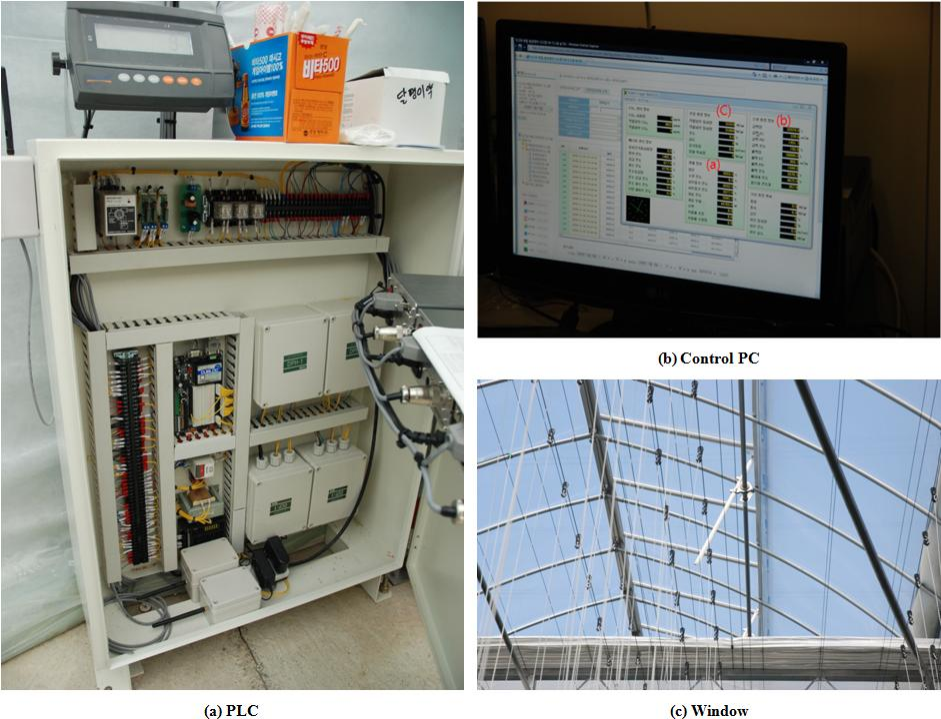

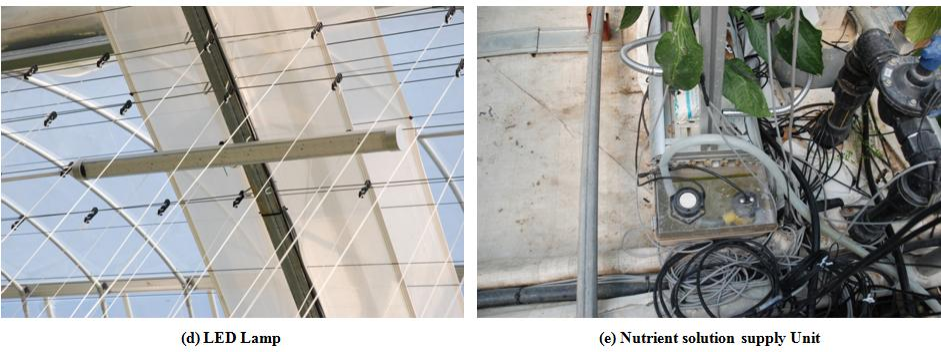
Environment Control Facilities and PLC.

In order to create an optimized paprika growth environment based on the crop growth information from the sensors installed inside and outside the greenhouse, weather condition information from inside and outside the greenhouse, and information about the rooting zone environment, environment control facilities were installed in the greenhouse, and in order to control these facilities, a PLC controller was installed. Figure 13 shows the installed environment control facilities and PLC in the paprika greenhouse.

**Figure 14.**
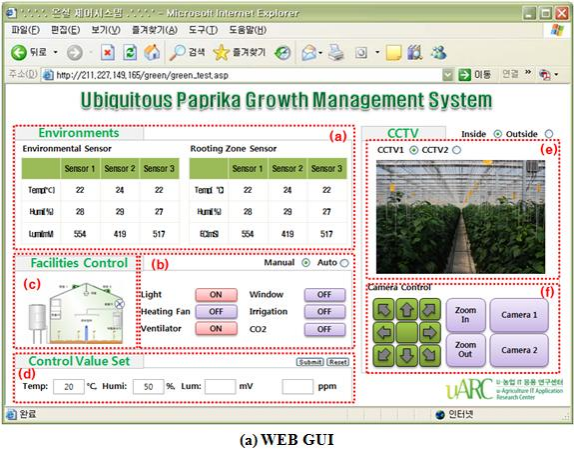

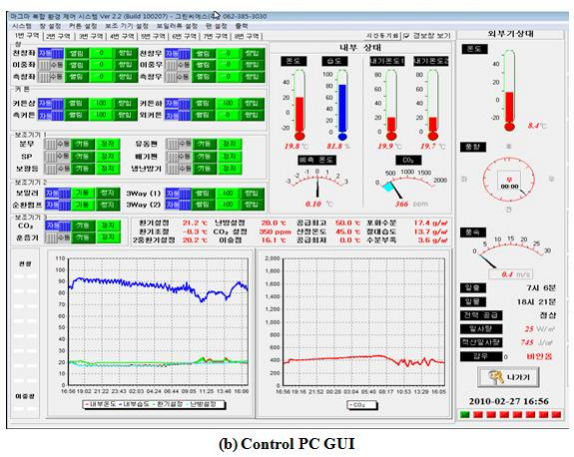
Ubiquitous Paprika Growth Management System GUI.

In order to monitor and control the paprika greenhouse, as seen in the Figure 14, a GUI for managers was developed as a web environment. WAS (Web Application Server) uses Tomcat-6.0.20 and the database Mysql 5.0 which is the safest version among the versions that are currently released.

In the manager GUI, the sensing values measured by the sensors installed inside and outside greenhouses appeared in (a), and (b) shows control of equipment in greenhouses and its conditions; (c) expresses the conditions of equipment in (b) as graphics; (f) is the part to control the CCTV system; (e) is the part to show images collected through the CCTV cameras, and (d) id the part to enter standard values for automatic greenhouse control.

**Figure 15.**
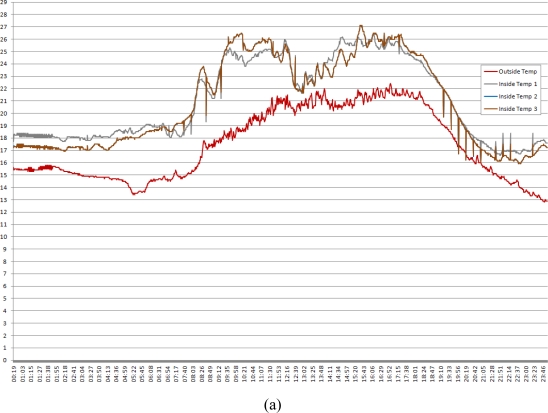

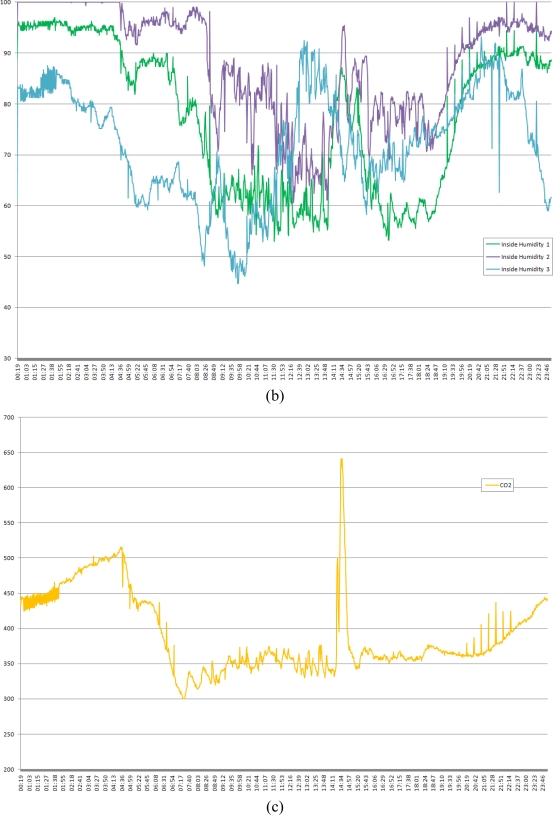

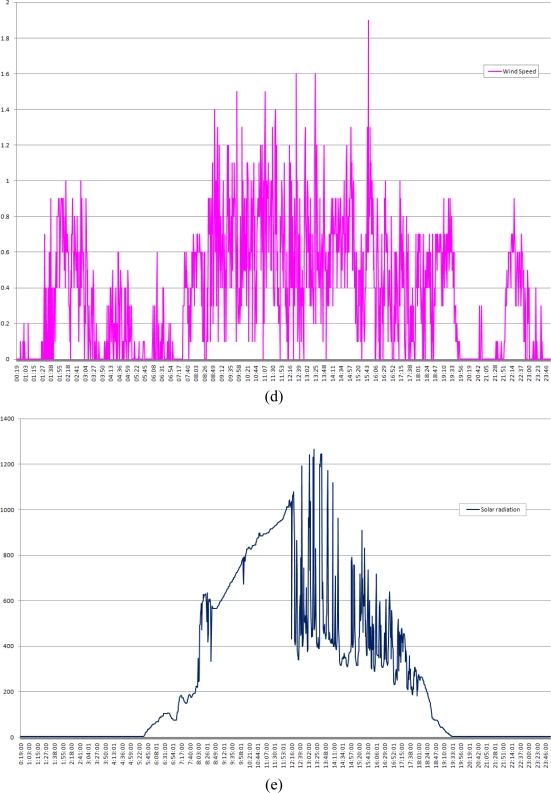
Measured Environmental Data Graph (1 June 2010). **(a)** Inside Temperature and Outside Temperature; **(b)** Inside Humidity; **(c)** CO_2_; **(d)** Wind Speed; **(e)** Solar Radiation.

As a result of applying the proposed system as mentioned above to actual paprika greenhouses, information about the environment and greenhouse images are collected through sensors and image supervision cameras, and the GUI, which is intuitive to users, can monitor and control greenhouse conditions. Figure 15 is a graph that shows growth and development data measured by installing the proposed ubiquitous paprika growth management system in an actual paprika greenhouse.

The monitoring values related to greenhouse control and the control values in the graphs above show that there were no operation errors. In addition, in monitoring rooting zone environment and information about the plants, there is a need for the consistent research and supplementation data that the sensor monitoring system can provide.

## 5. Conclusions

This study proposes a ubiquitous paprika growth system for comprehensive management of paprika greenhouses that require precise management of the plant growth and development environment. The proposed system is composed of physical, middle and application layers, and components of each layer collect and manage information of environment for growth and development within paprika greenhouses. Not only is the information delivered to users by a variety of methods, but remote manual and automatic control of paprika greenhouses also improves users’ convenience and productivity, and based on the data of environment for growth and development gained by operating the system, an optimized environment for growth and development of paprika can be created.

To test the proposed system, it was tested in paprika greenhouse models by installing sensors, including sensors for soils, environment, temperature and humidity of leaves and CCTV cameras and it was was established in actual paprika greenhouses. As a result of this testing, monitoring and control of the paprika growth and development environment could be conducted through a convenient GUI and the sensor monitoring showed there were no errors in the operation of the greenhouses.
